# The Derelict Child and the State

**Published:** 1921-07-30

**Authors:** 


### The Derelict Child and the State.

Lord Lytton again presided this year at the annual meeting, held on July 14, of the State Children's Association. This Society works away quietly to aid the child left to State care by loss of family or incipient criminality. It has scored a great victory lately in its campaign against the attempt of authorities in several places to collect the children from their various Unions into one workhouse from which the older paupers are removed, thereby reinstating the condemned system of barrack schools. All such schemes have now been dropped. But it is not satisfactory to hear that on January 1 there were 2,253 children over three years old in workhouses, though this is a decreased number. Forty-eight States and two Territories in America have now adopted the plan of widows' pensions; that is to say, of a maintenance allowance for each child of dependent age, which is actually more economical than the system we still follow of placing the children in costly institutions or paying other women to bring them up, while the mother faces the world alone and has neither home nor parental responsibility. The cost of deterioration in health and morals of mother and children is not the less real because not easily estimated. The Association gives its support to the Child Adoption Bill, which finally establishes a child in a real family, under proper precautions. Some of these, indeed, give food for grave thought. In view of the decision of so many young married people not to have children, adoptions are becoming more numerous in all classes; and while at present the greater number of the adopted are still small children, in less than a score of years we may expect a new crop of social problems.

The State Children's Association counts it a great gain that four Special Children's Courts, apart from Police Courts and shorn of all legal paraphernalia, have been started. They want, however, to see more done in inquiring as to the physical health of the incipient delinquent, and would welcome more use of policewomen. A matron takes charge, usually, of children awaiting hearing at any Police Court, but little girls, as well as boys, must enter in a policeman's charge, and when called as witnesses in ordinary courts may be entirely alone. Quite lately a child of six had to give evidence without even the presence of its mother. Indeed, the whole question of the evidence of children is a very serious one. One child may be terrified by its surroundings and by dread of the consequences of truth-telling. Another may be one of those fluent little liars who are so astonishingly consistent sometimes in an entirely imaginary or a prompted tale.

There is also much to be done with regard to children in prison, whether serving short sentences or detained for trial. The evidence of some conscientious objectors has been useful, and goes to show that segregation of children is by no means complete. Even upon the "young adult," sixteen to twenty-one, the breath of prison passes so harmfully that the girls tend to become prostitutes and the boys criminals—in prison they are taught no trade. Nor is the punishment of the rod, which seems to magistrates who have been to Public Schools so simple and quickly over, satisfactory in its results. One in four of birched boys return in a month, eighty per cent. within four years.

